# Supplementary material for: Metaphors that shape parents’ perceptions of effective communication with healthcare practitioners following child death: a qualitative UK study
Source: BMJ Open. 2022 Jan 24;12(1):e054991. doi: 10.1136/bmjopen-2021-054991 (PMC8796225; doi:10.1136/bmjopen-2021-054991)
Supplement: Author's manuscript [file bmjopen-2021-054991.draft_revisions.pdf]

BMJ Open is committed to open peer review. As part of this commitment we make the peer review history of every article we publish publicly available.

When an article is published we post the peer reviewers' comments and the authors' responses online. We also post the versions of the paper that were used during peer review. These are the versions that the peer review comments apply to.

The versions of the paper that follow are the versions that were submitted during the peer review process. They are not the versions of record or the final published versions. They should not be cited or distributed as the published version of this manuscript.

BMJ Open is an open access journal and the full, final, typeset and author-corrected version of record of the manuscript is available on our site with no access controls, subscription charges or pay-per-view fees (http://bmjopen.bmj.com).

If you have any questions on BMJ Open's open peer review process please email info.bmjopen@bmj.com

BMJ Open

Metaphors that shape parents' perceptions of effective communication with healthcare practitioners following child death: a qualitative UK study

Journal:	*BMJ Open*
Manuscript ID	bmjopen-2021-054991
Article Type:	Original research
Date Submitted by the Author:	01-Jul-2021
Complete List of Authors:	Turner, Sarah; Coventry University, Faculty of Arts and Humanities<br>Littlemore, Jeannette; University of Birmingham, English Language and Linguistics<br>Topping, AE; University of Birmingham College of Medical and Dental Sciences, School of Nursing; University Hospitals Birmingham NHS Foundation Trust,<br>Parr, Eloise; University of Birmingham Edgbaston Campus<br>Taylor, Julie; University of Birmingham, School of Nursing, College of Medical and Dental Sciences, ; Birmingham Women's and Children's NHS Foundation Trust,
Keywords:	Paediatric palliative care < PAEDIATRICS, Paediatric oncology < PAEDIATRICS, Paediatric A&E and ambulatory care < PAEDIATRICS

SCHOLARONE™
Manuscripts

BMJ

I, the Submitting Author has the right to grant and does grant on behalf of all authors of the Work (as defined in the below author licence), an exclusive licence and/or a non-exclusive licence for contributions from authors who are: i) UK Crown employees; ii) where BMJ has agreed a CC-BY licence shall apply, and/or iii) in accordance with the terms applicable for US Federal Government officers or employees acting as part of their official duties; on a worldwide, perpetual, irrevocable, royalty-free basis to BMJ Publishing Group Ltd ("BMJ") its licensees and where the relevant Journal is co-owned by BMJ to the co-owners of the Journal, to publish the Work in this journal and any other BMJ products and to exploit all rights, as set out in our licence.

The Submitting Author accepts and understands that any supply made under these terms is made by BMJ to the Submitting Author unless you are acting as an employee on behalf of your employer or a postgraduate student of an affiliated institution which is paying any applicable article publishing charge ("APC") for Open Access articles. Where the Submitting Author wishes to make the Work available on an Open Access basis (and intends to pay the relevant APC), the terms of reuse of such Open Access shall be governed by a Creative Commons licence – details of these licences and which Creative Commons licence will apply to this Work are set out in our licence referred to above.

Other than as permitted in any relevant BMJ Author's Self Archiving Policies, I confirm this Work has not been accepted for publication elsewhere, is not being considered for publication elsewhere and does not duplicate material already published. I confirm all authors consent to publication of this Work and authorise the granting of this licence.

For peer review only

Metaphors that shape parents' perceptions of effective communication with healthcare practitioners following child death: a qualitative UK study

Title Page

1 Dr Sarah Turner[1, *] sarah.turner@coventry.ac.uk Lecturer in English (Stylistics) https://orcid.org/0000-0003-3414-6828

2 Professor Jeannette Littlemore[2] j.m.littlemore@bham.ac.uk Professor of Applied Linguistics https://orcid.org/0000-0003-4670-0275

3 Professor Annie Topping[3,4 §] a.e.topping@bham.ac.uk Professor of Nursing https://orcid.org0000-0002-0111-2341

4 Eloise Parr[2] ebp969@student.bham.ac.uk Research Assistant https://orcid.org/0000-0002-7415-5991

5 Professor Julie Taylor[3,5] j.taylor.1@bham.ac.uk Professor of Child Protection https://orcid.org/0000-0002-7259-0906

[1]School of Humanities, Coventry University, Coventry, UK

[2]Department of English Language and Linguistics, University of Birmingham, Birmingham, UK

[3] School of Nursing, University of Birmingham, Birmingham, UK

[4] University Hospitals Birmingham NHS Foundation Trust, Birmingham, UK

[5]Birmingham Women's and Children's Hospitals NHS Trust, Birmingham, UK

*Lead author address: School of Humanities, Coventry University, Coventry, UK

§ Corresponding author address: College of Medical and Dental Science, University of Birmingham, Edgbaston, B15 2TT, UK

For peer review only - http://bmjopen.bmj.com/site/about/guidelines.xhtml

Metaphors that shape parents' perceptions of effective communication with healthcare practitioners following child death: a qualitative UK study

Abstract

Objectives To offer an interpretation of bereaved parents' evaluations of communication with healthcare practitioners surrounding the death of a child.

Design Interpretative qualitative study employing thematic and linguistic analyses of metaphor embedded in interview data.

Setting England and Scotland.

Participants 24 bereaved parents (21 women, 3 men)

Methods Participants were recruited through the True Colours Trust website and mailing list, similar UK charities, and word of mouth. Following interviews in person or via video-conferencing platforms (Skype/Zoom), transcripts first underwent thematic and subsequently linguistic analyses supported by Nvivo. A focused analysis of metaphors used by the parents was undertaken allow in-depth interpretation of how they conceptualised their experiences.

Results The findings illuminate the ways parents experienced communication with healthcare practitioners surrounding the death of a child. They show how their evaluations of effective care relate to the experience of the bereavement itself, as expressed through metaphor. We identified three broad themes: (1) Identity (2) Emotional and Physical Response(s) and (3) Time. Successful communication from healthcare practitioners takes account of parents' experiences related to these themes.

Conclusions This study suggests that healthcare practitioners when communicating with bereaved parents need to recognise, and seek to comprehend, the ways in which the loss impacts upon an individual's identity as a parent, the 'physical' nature of the emotions that can be unleashed, and the ways in which the death of a child can alter their metaphorical conceptions of time.

Keywords bereavement, communication, metaphor, child death, healthcare professionals, parents

Strengths and limitations of the study

- This interpretative qualitative study contributes to the growing literature on the experience of parental bereavement through its focus on the use of metaphor in parents' accounts of child loss.

For peer review only

- The metaphor analysis afforded a focus not only on the content of the interviews, but also on the language that was used to express this content, providing more depth and nuance to the interpretation of the findings.
- Participants were all UK-based and self-selecting which could limit transferability.

For peer review only

For peer review only - http://bmjopen.bmj.com/site/about/guidelines.xhtml

Metaphors that shape parents' perceptions of effective communication with healthcare practitioners following child death: a qualitative UK study.

INTRODUCTION

When parents experience the death of a child, no matter the age, they experience a range of complex, often intolerably painful emotions[1]. The experience and effects of the death of a child on parents has received considerable attention, including the psychosocial impacts experienced as a consequence of devastating and enduring grief[2]; the experience of parents around end of life of a child[3–5]; what they want and expect from healthcare practitioners (HCPs)[2,6]; and impacts of bereavement services and interventions[1,7]. Less work has focused on the communication remembered by parents as part of their experiences of care or services received, or how that communication subsequently scaffolds their experience of bereavement. However, negative interactions with HCPs following the death of a child have been shown to exacerbate the trauma experienced by the parents[8], while positive interactions such as empathy and provision of information can mitigate it[9,10]. A better understanding of the impact of bereavement and the ways in which it affects parents' evaluations of the communication they had with HCPs in the period surrounding the death may enable design of structures, processes, interventions and training to improve individualised compassionate bereavement care.

BACKGROUND

The death of a child engenders a range of intense and complex emotions that may be difficult to articulate[11]. The loss of a child has been associated with poorer parental physical health and increased mortality[12]. Staff regularly providing end-of life care also experience high levels of emotional exhaustion[5]. These are further compounded when HCPs feel guilt about inadequate, or missed care, poor communication and/or contain expressions of grief or emotions in order to protect families from further distress[13]. Effective communication following the death of a child is therefore important for both parents and HCPs.

Staff involved in managing deaths occurring in healthcare settings have well practiced roles and responsibilities. Sociocultural values and beliefs about death and dying are implicitly embedded in the organisation of services and inevitably demonstrated in the behaviours, attitudes and discourses used by the staff involved[14]. This is most evident in what could be interpreted as a proceduralisation of dying[15,16] which can exclude the individual who is dying and parents and family members experiencing the loss. The well rehearsed concept of a good death is less well understood in relation to the death of a child or young person[17]. Possibly as greater emphasis is placed on dying and/or bereavement rather than the period surrounding death, and different participants (patients, family/parents, and staff) may have different needs and requirements for control and participation/non-participation in the social context where the dying and death are enacted.

For peer review only - http://bmjopen.bmj.com/site/about/guidelines.xhtml

Parents provide mixed accounts of the care that they recall having received in the period surrounding the death of their child[2,18] with some reporting highly positive experiences of compassionate care and others less positive accounts. What is clear is parents' value open and honest communication with HCPs, want to be actively involved, and their emotional distress acknowledged. Ideally they want to communicate with HCPs with whom they have already developed established and trusting relationships[4]. Furthermore, they want to say goodbye, to understand why their child died and to receive follow up care from HCPs involved in the care of their child[2]. Ensuring these standards are achieved may be particularly challenging in the case of sudden death or in emergency, trauma and critical care contexts. This means that good guidance for healthcare practitioners on how best to communicate with parents following the death of their child is essential.

In this study, we focused on the experiences of parents in the immediate aftermath of the death of their child, and their recollected communications with HCPs. We included parents of both adult and non-adult children. The term 'healthcare practitioners' (HCPs) is used in a broad sense to include all of those who come into contact with the parents at the hospital, not just the doctors and nurses.

METHODS

Aim

The aim of this research was to investigate how parents' experiences of bereavement and child death shaped their perceptions of the communication they had with HCPs. In this paper, we consider 'communication' to encompass both language and behaviour, and the interactions between actors.

By gaining insights into parents' experiences of child death, we sought to better understand what constitutes effective communication, with a view to improving care after loss.

Design and Setting

We used an interpretative descriptive qualitative design[19]. The study is reported using Consolidated criteria for Reporting Qualitative research guidelines (see online supplementary file).

Participants

Our participants comprised parents aged at least 18 years who had lost a child of any age, who could speak English at conversational level, and who were able to provide informed consent.

Sampling and recruitment

A purposive sampling procedure was employed. Recruitment adverts were posted by the True Colours Trust, Compassionate Friends, and other similar charities (e.g. Acorns Children's Hospice, Winston's Wish etc). Potential participants who responded to the posting were contacted by the research team and invited to participate in an interview.

For peer review only - http://bmjopen.bmj.com/site/about/guidelines.xhtml

Data collection

In total, 24 participants were interviewed, in 21 interviews. The majority of the interviews were conducted on a 1-1 basis, with all authors undertaking interviews. On three occasions however we undertook joint interviews with male and female participants at their request (interviews 2, 5 and 20). All interviews involved female participants (n=21). Interestingly just under half of our respondents were themselves HCPs. The interviews were conducted face to face (n=8), or via video-conference platforms (n= 13), according to participant preference, location, or COVID-19 restrictions. All but two of the face to face interviews were conducted in participants' homes, except two conducted at the Charity headquarters. Interviews lasted an average of 81 minutes (range: 34-129 mins).

Prior to interview, participants were informed how the findings derived from their interview data would be reported and disseminated in training materials. All participants provided informed consent prior to interview. Interviews were undertaken by all five authors, with some interviewers working together. A topic guide was developed to serve as a framework for the semi-structured interviews (see box 1). Open questions were used to encourage extended answers, and to allow the interviewer(s) to elicit further detail on points of interest.

Box 1 Sample of interview prompts

- Can you tell us about [name of child]?
- Please could you describe what happened ….?
- Please could you tell me about what happened next…?
- How did you feel about…?
- Can you tell me about the communication you had with the healthcare practitioners, funeral directors and registrars?
- Can you recall any examples of particularly sensitive and effective communication you received?
- Can you recall any examples of particularly insensitive communication you received?
- Imagine you are talking to someone who supports people who have had an experience like yours. What advice would you give them?
- What would you like to see changed?

Audio recordings were made of the interviews with consent. In the case of video-conference interviews, recordings included video data, but these were destroyed and only audio recordings retained.

Analysis

Audio recordings were transcribed verbatim and all identifying information removed. Transcripts were sent to the participants to allow them to make any corrections or additions they felt necessary. Interview transcripts were coded using NVivo.

We first undertook a thematic analysis to identify parents' experiences of bereavement and communication. A full list of identified themes is provided in the online supplementary file 1].

For peer review only - http://bmjopen.bmj.com/site/about/guidelines.xhtml

Subsequently, we combined our thematic analysis with a metaphor analysis which enabled us to explore how parents conceptualised these experiences. The metaphor analysis involved coding the language used by participants relating to each of the categories for metaphor. This focus on metaphors was designed to provide deeper insights into the nature of the experience, and explore the extent to which parents' perceptions of communication could be explained by these experiences.

In order to identify metaphor in the transcripts, we used Cameron and Maslen's Vehicle Identification Procedure[20]. This analytic procedure operates at the level of the phrase and involves recognition of the fact that something is being described in terms of a different entity.

Patient and Public Involvement

The study engaged with bereaved families throughout. It was designed in communication with the True Colours Trust who funded the research, and who work closely with bereaved parents. Study results will be shared with all participants and ultimately integrated in training materials.

FINDINGS

Participants

Twenty-four people participated in 21 interviews. Table 1 gives further information about the circumstances of death in each interview.

Table 1: Circumstances of death

Interview number	Age of child (years)	Cause of death	When death occurred
1	0-5	RTC	<5yrs
2	0-5	Illness (sudden)	>5yrs
3	16-20	Illness (sudden)	>5yrs
4	0-5	Illness (sudden)	>5yrs
5	16-20	Drug-related death	<5yrs
6	0-5	Illness (sudden)	<5yrs (1)
7*	0-5	Illness (genetic condition)	>5yrs + <5yrs
8	0-5	Illness (sudden)	>5yrs
9	16-20	Suicide	>5yrs
10	21-30	Suicide	>5yrs

For peer review only - http://bmjopen.bmj.com/site/about/guidelines.xhtml

11	16-20	Drug-related death	<5yrs
12	21-30	Unknown (ongoing inquiry)	<5yrs
13	21-30	Suicide	<5yrs
14	21-30	Illness (cancer)	<5yrs
15	16-20	RTC	>5yrs
16	21-30	Murder	<5yrs
17	2mo	Illness (sudden)	<5yrs
18	17days	Illness (sudden)	>5yrs
19	22days	Illness (genetic condition)	>5yrs
20	17mo	Illness (sudden)	<5yrs
21	2yrs	Illness (sudden)	>5yrs

* interviewee discusses two child deaths associated with the same inherited condition

In all our interviews, we found that the parents had vivid memories of some parts of the peri-death experience whereas others were more hazy. In all cases, the ways in which HCPs communicated with the parents during this period played a crucial role in shaping the experience. In this example, the parent uses metaphor to illustrate the painful impact of the doctor's words:

> years later, I can remember every word. I can even remember his tone of voice as he said it. Absolutely etched with acid into my brain
> [Interview 3]

Through our thematic and metaphorical analyses we identified 14 categories, which included different kinds of communication, people involved, and experiences surrounding events associated with the loss (e.g. funerals). These are shown in Table 1 in the supplementary file.

To answer our research questions we examined the metaphors within these categories in order to identify overarching themes in the data where metaphor was playing an important role. The three themes reported here and shown in Figure 1 are: (1) Identity (2) Emotional and Physical Response and (3) Time.

[Figure 1]

Figure 1: Metaphorical Themes

For peer review only - http://bmjopen.bmj.com/site/about/guidelines.xhtml

Identities of parents and children

Parents appeared to struggle with the loss not only as the death of the child but also as a significant threat to their identities as parents. Some parents talked about how leaving their child in the hospital after death was made more difficult, as it constituted ‘leaving them behind’. Parents felt that they still needed to look after their child, as one parent explained how she was ‘distraught’ that her daughter was ‘shut up’ in the mortuary as she was claustrophobic. These utterances reflect a level of metaphoric thinking in which parents conceptualise their child as being, on some level, still alive. Here a bereaved parent talked about the excellent care received when they went to identify their son’s body:

> [a member of staff at the mortuary] crouched down, she took my hand and said ‘hello NAME, I’ve got your little boy and I’m keeping him safe, do you want to come and see him?’ [Interview 5]

Many of the parents commented on how they were given the time and space to act in ways that seemed right to them as it respected their child’s identity and theirs as parents. One couple (Interview 2) appreciated how the consultant arranged for their child’s body to be taken to a children’s hospice, where it could be kept in a refrigerated room that had been decorated like a child’s bedroom.

Conversely, some parents reported examples of care and communication that failed to recognise their child as an individual. This parent describes the distress she felt when hospital staff attempted to fit her 18-month-old child into a Moses basket:

> They tried to put him in a Moses basket cos they thought it was a cot death, they obviously didn’t say the age on the phone call across [...] They probably should know about the age of the child, that was really – cos they did try and like squeeze him in in a way as well. So even when – you could clearly see he wasn’t gonna fit in that. I just thought that was quite upsetting. Like he wouldn’t have been in a Moses basket at that age anyway [Interview 2].

This was particularly distressing to the parents because it seemed that the HCPs were more concerned with following the procedure rather than with responding flexibly to the reality of the situation. This contributed to the parents’ impression that the care their child received was a “one size fits all” rather than tailored to the individual needs of their child.

In some cases, parents were told that they were not allowed to interact with their child following the death because the circumstances of the death were unknown. Although necessary, it caused extreme distress, exacerbated by insufficient explanations and/or management of parental expectations. Another couple (Interview 6) spoke of the ‘horrific’ experience of having to take a service lift down to the ‘old-fashioned’ and ‘cold’ mortuary. The parents felt that they were insufficiently prepared for what they would encounter, and their role as parents was discounted to that of observers of a body.

For peer review only - http://bmjopen.bmj.com/site/about/guidelines.xhtml

Emotional and Physical Responses

A second theme related to the 'physical' nature of emotions unleashed by the death of a child. Parents often referred to these experiences in metaphorical ways conveying the strength of their emotional response to the death.

> You're frozen, you're like a zombie, you just can't, everything's frozen up inside you [Interview 3]
>
> It's like you're full of cotton wool in your head and people do have to keep telling you things over and over and over again … so just kind of time, don't be surprised you've got a woolen brain sitting in front of you
>
> [Interview 3]

These quotes emphasise the effect death has and how HCPs may need to recognise this and in terms of ability of parents to receive information. In the following example, a parent talks about how she had kept a bereavement support leaflet from the hospital for some months following her daughter's death.

> It was there in the booklet that HOSPITAL had given me [...] my attention was directed to it and I hadn't noticed it. I'd been to my doctor and he'd given me sleeping tablets and I wasn't taking them, I was saving them up. It was stupid, I know it was stupid. [I was] sitting with all these tablets and a bottle of wine and just thinking, 'I can't do this'. … And I thought I'm gonna have to get help and I knew the Samaritans number was in that booklet they'd given me. So I'd found the booklet, found the Samaritans and then just underneath I saw Compassionate Friends. And so I phoned the helpline … and got an answerphone, 'We'll get back to you' and I thought, 'I bet they don't', but they did. And I spoke to a lady called NAME ... and she was great. She'd lost her daughter about 14 or 15 years before and just hearing her say that she'd survived more than anything else helped me.
>
> [Interview 3]

At times parents employed metaphor to express the fact that they no longer wanted to engage with the world:

> We just wanted to hide. Literally had this sense of 'I wanna get a cave, I don't wanna see the world, I don't wanna engage with anybody or anything'. I can't function with the world being normal. I nearly always wore contact lenses, I rarely wore glasses but [then] I wore glasses on purpose so that I could just take them off half the time, cos I just wanted everything to go away [Interview 4].

She draws a metaphorical comparison between the act of seeing and the act of engaging with the world around her, which allows her to take control of her situation by literally removing her glasses, thus enabling her to metaphorically withdraw from the world.

At times, parents commented that HCPs appeared not to take sufficient account of the fact that appearing calm concealed the depth of their distress, how staff mistook calmness for strength

For peer review only - http://bmjopen.bmj.com/site/about/guidelines.xhtml

and indeed congratulated parents for coping. Whereas the bereaved felt under pressure to present a 'brave face':

> Throughout the early days people (hospital staff etc.,) would constantly say how strong and brave I was. I kept saying " no I'm not, I'm just pretending", but eventually I felt that I had to be this "brave, strong" woman or I would be letting everyone down. If I could say one thing to them [hospital staff] it would be to ask them not to say that. We are not brave and strong, just in a state of severe shock and watching ourselves go through the motions and it's really unhelpful to make us feel we must behave in a certain way [Interview 11].

By praising parents HCPs were inadvertently reinforcing the idea that following bereavement, some kinds of behaviour tend to be more acceptable than others and that overt emotional displays are to be discouraged[4].

Time

Previous studies have shown that trauma can exert a strong effect on perceptions of time, reporting the phenomena of time stopping, slowing down, speeding up or becoming irrelevant[21]. All of these descriptions draw on the metaphorical relationship between time and movement through space[22]. Time appeared to be experienced differently by parents on the death of a child, and time becomes foregrounded in their experience. This is demonstrated in the following examples:

> So suddenly … I remember thinking, 'Slow down! It's like I still don't know what's happened,' and then suddenly, we were like, 'Whoo!' and we were heading towards an inquest but having said that, it was a year, this whole process ... so it wasn't slow down time. It was just - so we had no control [Interview 1].

> when you've had such a massive shock like that, it just takes so long for your brain to catch up [Interview 1].

Disordered time appeared to influence how interactions with HCPs were perceived. For example what could be viewed as acts of caring for were interpreted as simply procedural and are therefore less effective. In some cases, this may prevent parents from reaping any benefit from the care provided. For example, one participant described receiving a memory box without any (remembered) explanation of what it was, or what was in it, and did not dare to open it:

> At that point, a nurse put something in my mum's hands and it was inside a cardboard box and just said, 'Here's his memory box'. So my mum was a bit like, 'Thanks'. It was horrific for her. So she walked out, there was no other explanation of it and we walked out and she just put it on the dining table when we got home. And like for days after everyone was like, 'What's in that box?', but no one dared open it because - my mum was saying, 'They said oh it's his memory box, I didn't want to open it in case it's his things in there' [Interview 6].

We see from this example that what may have started out as an enactment of good practice in bereavement support - the giving of a memory box - had become for this parent, something to be feared due to the way in which it was presented.

For peer review only - http://bmjopen.bmj.com/site/about/guidelines.xhtml

For peer review only

DISCUSSION

The findings from this study suggest that good communication with HCPs following the death of a child should acknowledge parental identity (and that of their child as an individual) and offer opportunities for them to enact this; taking account their emotional and physical experiences; and accommodate their altered experiences of time. Practitioners need to acknowledge the individual needs of the parents while working within the parameters of best practice and legal considerations[16,23].

Identity

In keeping with previous research[24,25], we found that many of the parents reported a strong and sustained bond with their child, which led to a continuing need to 'parent' their child. The importance of these bonds may be understood as a response to the threat to parental identity that the loss of a child represents. Thus parents experiencing their child's death are in part losing their role as a parent [26]. Parents in our study highlighted the importance of being able to spend time with their child following death and to continue enacting their roles as parents[27–29]. The opportunity to do this allows for a 'slow and gentle separation' at a pace set by the parents, reduced feelings of helplessness, and the potential to form 'bittersweet memories' which may later represent a source of comfort[27,28]. The importance of protected time within the grief process particularly spending time with the child's body represents symbolically privileged status of parent and other family members in death[30].

The behaviour of parents towards their child's body can be understood as an example of metaphorical thinking, where the child is perceived as needing the same care as in life. This involves a blurring of the boundaries between life and death where parents enact behaviours they would have engaged in if the child were alive. Our study provides further evidence of the importance of opportunities to engage in these behaviours, and their facilitative role in the grieving process.

HCPs have an important role to play in acknowledging bereaved parents' continuing bonds with their children, and facilitating opportunities for parents to perform these behaviours. The parents in our study made frequent references to cases where HCPs were either successful in doing so, or where they felt the care they received was lacking in this regard. This was often a determining factor in their evaluation of the effectiveness of the overall care that they had received. For example, parents in our study appreciated instances of HCPs treating their child's body with respect and dignity. This was represented in our findings describing HCPs speaking to children after death as a means of respecting the child's presence and personhood[31]. Effective linguistic and behavioural communication therefore involves recognising that the loss of a child represents a significant threat to parental identity, and that parents should be given the time, space and opportunity to engage in activities that enable them to enact that identity. Similarly, there is a need to understand the continuing bonds that the parents still have with their child, even when these may be appear in ways that seem unusual.

Emotional and physical response

The parents in our study drew heavily on embodied metaphors to describe the strength and impact of the bereavement and ensuing emotions. The metaphors used had such a strong

For peer review only - http://bmjopen.bmj.com/site/about/guidelines.xhtml

physical basis is in line with other studies that propose powerful, embodied metaphors can be the only device strong enough to to express emotions following traumatic experiences[32,33,33–36]. Although metaphor is sometimes seen as a poetic device, operating solely at the level of language, we have demonstrated that it is inappropriate to see it as a surface-level linguistic phenomenon, particularly in the context of traumatic experiences. The physical nature of the metaphors that bereaved parents employ when describing the intensity of their experiences and the fact that these metaphors sometimes spill over into their behaviour (e.g. the parent who literally removed her glasses) provide powerful insights into their physical and emotional state and as such are not simply 'figures of speech'. These observations are also in line with previous research that has demonstrated that grief and emotional trauma may manifest as physical sensations[37].

Effective care and communication following child death takes account of the all-encompassing, frequently embodied nature of grief, and recognises that this may have an effect on parents' abilities to make decisions and understand the meaning communicated in the care provided. Grief may manifest in behaviours that appear unusual or may go unnoticed (removing glasses), but are motivated by highly individualised metaphorical thinking patterns that represent the parents' attempts to manage and come to terms with their experiences.

Time

Following the death of a child, parents may perceive or experience time differently. Time might metaphorically expand or contract, or do both at the same time, and parents may feel that they are no longer part of the same 'reality' as those around them. While altered perceptions of time over the course of the grieving process have been attested to in narratives following child loss[38], our findings indicate that changed experiences of time in the immediate aftermath of a loss may also have important implications for care. It is therefore important for HCPs to recognise that parents' emotional state may not be conducive to receiving and processing complex information, and they may need to be given the information more than once and/or in more than one format[32]. HCPs should demonstrate patience and sensitivity to parents' difficulties in processing information.

There is a danger that care following the death of a child can become procedural, and as a result may not be empathetic to the needs of parents. For example, providing a memory box may constitute excellent bereavement care, but if this is given without clear explanation, or choice, may become a source of anxiety. . Following formal procedures without sensitivity to individual needs may result in greater disquiet.

This study has contributed to the growing literature on the experience of parental bereavement but its focus on the use of metaphor in parents' accounts of child loss provides valuable insights. Key aspects of the experience have been identified that need to be considered when providing care for the bereaved. Our metaphorical analysis has afforded a focus on the on the language that was used to express content in qualitative interviews, providing more depth and nuance to interpretation. The number of participants was relatively small UK-based and self-selecting which could limit the transferability of our findings. Future studies could usefully explore metaphor with more participants with different demographic characteristics and in different settings.

For peer review only - http://bmjopen.bmj.com/site/about/guidelines.xhtml

CONCLUSION

This study has shown how the analysis of metaphors used by parents when describing their experiences of bereavement can provide a deep and nuanced understanding of their experiences and how these experiences affect their evaluations of the communication they receive from HCPs. We have demonstrated that effective communication and strategies to ameliorate distress entails an understanding of the feelings and experiences of the bereaved and takes these into account. Conversely, ineffective communication is not grounded in this awareness.

Effective communication that takes account of parents' experiences of bereavement may involve:

Identity:

- Demonstrating an awareness and acknowledgement of the parents' identities as parents, and the child's personhood
- Demonstrating respect for the child's body and reassure parents that the child is being cared for even after death
- Facilitating opportunities for the parents to enact their roles as parents

Emotional and Physical Responses

- Being aware that the all-encompassing nature of the grief may render parents unable to process and act upon information
- Recognising that parents may engage in individualised behaviours that may seem unusual or go unrecognised as enactments of grief.

Time

- Recognising that parents may be experiencing time in altered ways, and that this may have an impact on their ability to process information.
- Explaining information in a clear but sensitive way, recognising that information may need to be given more than once or in different formats.
- Demonstrating patience, giving parents time and not rushing them through the various procedures or difficult decisions.

References

1 Donovan LA, Wakefield CE, Russell V, *et al.* Hospital-based bereavement services following the death of a child: a mixed study review. *Palliative medicine* 2015;**29**:193–210.

2 Garstang J, Griffiths F, Sidebotham P. What do bereaved parents want from professionals after the sudden death of their child: a systematic review of the literature. *BMC pediatrics* 2014;**14**:1–17.

For peer review only - http://bmjopen.bmj.com/site/about/guidelines.xhtml

3 Ekberg S, Bradford N, Herbert A, *et al.* Healthcare users' experiences of communicating with healthcare professionals about children who have life-limiting conditions: a qualitative systematic review protocol. *JBI Evidence Synthesis* 2015;**13**:33–42.

4 Ekberg S, Bradford NK, Herbert A, *et al.* Healthcare users' experiences of communicating with healthcare professionals about children who have life-limiting conditions: a qualitative systematic review. *Journal of palliative medicine* 2018;**21**:1518–28.

5 McConnell T, Porter S. The experience of providing end of life care at a children's hospice: a qualitative study. *BMC palliative care* 2017;**16**:1–6.

6 Tenzek KE, Depner R. Still searching: a meta-synthesis of a good death from the bereaved family member perspective. *Behavioral Sciences* 2017;**7**:25.

7 Kochen EM, Jenken F, Boelen PA, *et al.* When a child dies: a systematic review of well-defined parent-focused bereavement interventions and their alignment with grief-and loss theories. *BMC palliative care* 2020;**19**:1–22.

8 Janzen L, Cadell S, Westhues A. From death notification through the funeral: Bereaved parents' experiences and their advice to professionals. *Omega-Journal of Death and Dying* 2004;**48**:149–64.

9 Meert KL, Briller SH, Myers Schim S, *et al.* Examining the needs of bereaved parents in the pediatric intensive care unit: a qualitative study. *Death studies* 2009;**33**:712–40.

10 Price J, Jordan J, Prior L, *et al.* Living through the death of a child: A qualitative study of bereaved parents' experiences. *International journal of nursing studies* 2011;**48**:1384–92.

11 Arnold J, Gemma PB. The continuing process of parental grief. *Death studies* 2008;**32**:658–73.

12 Endo K, Yonemoto N, Yamada M. Interventions for bereaved parents following a child's death: A systematic review. *Palliative Medicine* 2015;**29**:590–604.

13 Papadatou D, Bellali T, Papazoglou I, *et al.* Greek nurse and physician grief as a result of caring for children dying of cancer. *Pediatric nursing* 2002;**28**:345–54.

14 Cheek J. At the margins? Discourse analysis and qualitative research. *Qualitative health research* 2004;**14**:1140–50.

15 Cottrell L, Duggleby W. The" good death": An integrative literature review. *Palliative & supportive care* 2016;**14**:686.

16 National Institute of Clinical Excellence. End of life care for infants, children and young people with life-limiting conditions: planning and management NICE guideline. 2016.www.nice.org.uk/guidance/ng61

17 Chong PH, Walshe C, Hughes S. Perceptions of a good death in children with life-shortening conditions: an integrative review. *Journal of palliative medicine* 2019;**22**:714–23.

For peer review only - http://bmjopen.bmj.com/site/about/guidelines.xhtml

18 Butler A, Hall H, Willetts G, *et al.* Parents' experiences of healthcare provider actions when their child dies: an integrative review of the literature. *Journal for specialists in pediatric nursing: JSPN* 2014;**20**:5–20.

19 Thorne S. Interpretative Description. In: Beck CT, ed. *Routledge International Handbook of Qualitative Nursing Research*. Routledge 2013. 295–306.

20 Cameron L, Maslen R. Metaphor analysis. *London: Equinox* 2010.

21 Holman EA, Grisham EL. When time falls apart: The public health implications of distorted time perception in the age of COVID-19. *Psychological trauma: theory, research, practice, and policy* 2020.

22 Moore KE. The spatial language of time. *Amsterdam & Philadelphia: John Benjamins* 2014.

23 National Quality Board. Learning from Deaths Guidance for NHS trusts on working with bereaved families and carer. https://www.england.nhs.uk/wp-content/uploads/2018/08/learning-from-deaths-working-with-families-v2.pdf

24 Harper M, O'Connor R, Dickson A, *et al.* Mothers continuing bonds and ambivalence to personal mortality after the death of their child–an interpretative phenomenological analysis. *Psychology, Health & Medicine* 2011;**16**:203–14.

25 Toller PW. Bereaved parents' negotiation of identity following the death of a child. *Communication Studies* 2008;**59**:306–21.

26 Klass D. *Parental grief: Solace and resolution*. Springer Publishing Co 1988.

27 Davies R. Mothers' stories of loss: their need to be with their dying child and their child's body after death. *Journal of Child Health Care* 2005;**9**:288–300.

28 Forrester L. Bereaved parents' experiences of the use of 'cold bedrooms' following the death of their child. *International journal of palliative nursing* 2008;**14**:578–85.

29 McGraw SA, Truog RD, Solomon MZ, *et al.* "I was able to still be her mom" Parenting at end of life in the PICU. *Pediatric critical care medicine: a journal of the Society of Critical Care Medicine and the World Federation of Pediatric Intensive and Critical Care Societies* 2012;**13**:e350.

30 Norton EA, Mastroyannopoulou K, Rushworth I. Parents experience of using "cold" facilities at a children's hospice after the death of their baby: A qualitative study. *Death Studies* 2020;:1–7.

31 Forster EM, Windsor C. Speaking to the deceased child: Australian health professional perspectives in paediatric end-of-life care. *International journal of palliative nursing* 2014;**20**:502–8.

32 Littlemore J, Turner S. What Can Metaphor Tell Us About Experiences of Pregnancy Loss and How Are These Experiences Reflected in Midwife Practice? *Frontiers in Communication* 2019;**4**:42.

For peer review only - http://bmjopen.bmj.com/site/about/guidelines.xhtml

33 Turner S, Littlemore J, Fuller D, *et al.* The production of time-related metaphors by people who have experienced pregnancy loss. *Producing Figurative Expression: Theoretical, experimental and practical perspectives* 2020;**10**:389.

34 Gibbs RW, Franks H. Embodied metaphor in women's narratives about their experiences with cancer. *Health communication* 2002;**14**:139–65.

35 Semino E. Descriptions of pain, metaphor, and embodied simulation. *Metaphor and Symbol* 2010;**25**:205–26.

36 Semino E. Metaphor, creativity, and the experience of pain across genres. *Creativity in language and literature: The state of the art* 2011;:83–102.

37 Gilmer MJ, Foster TL, Vannatta K, *et al.* Changes in parents after the death of a child from cancer. *Journal of pain and symptom management* 2012;**44**:572–82.

38 Barak A, Leichtentritt RD. Configurations of time in bereaved parents' narratives. *Qualitative Health Research* 2014;**24**:1090–101.

For peer review only

ACKNOWLEDGEMENTS

The authors thank the True Colours Trust for supporting this project.

CONTRIBUTORS

All authors contributed equally to all stages of the process.

COMPETING INTERESTS

None declared

FUNDING

This project was funded by the True Colours Trust

PATIENT CONSENT FOR PUBLICATION

Obtained

ETHICAL APPROVAL

The study had full ethical approval from University of Birmingham's Ethical Review Committee (ERN_19-1582)

For peer review only - http://bmjopen.bmj.com/site/about/guidelines.xhtml

PROVENANCE AND PEER REVIEW

Commissioned and funded by the True Colours Trust; grant number TCT1609; externally peer reviewed

DATA AVAILABILITY STATEMENT

No data are available. The research involved interviews in which bereaved parents gave personal accounts of their experiences. Participants consented to their data being used for this project only. Therefore, we will not share the data for any other purpose.

For peer review only

For peer review only - http://bmjopen.bmj.com/site/about/guidelines.xhtml

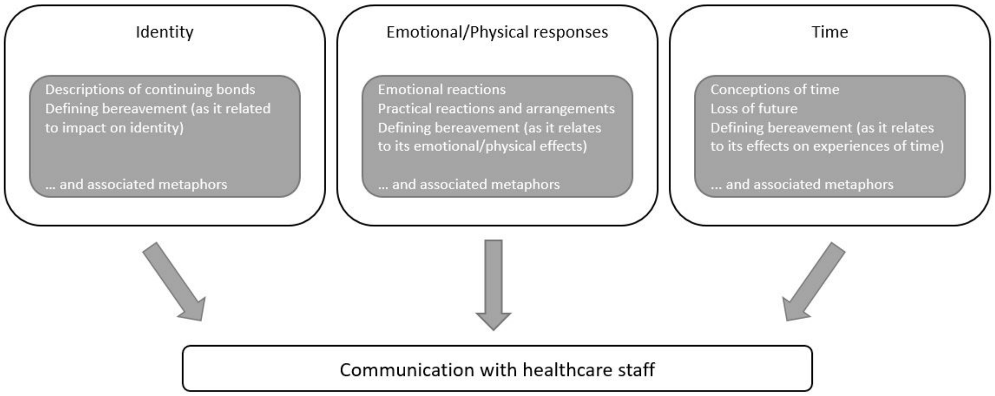

Figure 1: Metaphorical Themes

97x38mm (300 x 300 DPI)

Supplementary material Table 1

Coding categories used in our analysis

01 Bad Communication
- a Dehumanisation of child
- b Lack of empathy
- c Use of jargon
- d Ineffective use of time
- e Wrong information provided
- f Bad advice
- g Failure to communicate key information

02 Good Communication
- a Recognition of child
- b Empathy
- c Clear language
- d Effective use of time
- e Correct information provided
- f Good advice
- g Successful communication of key information

03 Emotional Reactions
- Emotional reactions - negative
- Emotional reactions - neither positive or negative
- Emotional reactions - positive
- Unexpectedly powerful reactions - negative
- Unexpectedly powerful reactions - positive
- Wished for emotional reaction

04 Practical Reactions and Arrangements
- Practical reactions and arrangements - neither positive or negative
- Practical reactions and arrangements - positive
- Practical reactions and arrangements - negative
- Symbolic Behaviours
- Unexpectedly powerful reactions - negative
- Unexpectedly powerful reactions - positive
- Wished for practical reaction

05 People Involved
- GPs
- Hospital staff
- Doctors
- Nurses
- Others - hospital
- Receptionists
- Mortuary staff
- Paramedics

06 Continuing Bonds

07 Loss of Future

08 Defining Bereavement

09 Conceptions of time

For peer review only

10 Metaphors
11 Advice that parents would give

For peer review only

Parents' experiences of healthcare professional communication following the death of a child: A qualitative study in the UK

Consolidated criteria for reporting qualitative studies (COREQ): 32-item checklist

Developed from:
Tong A, Sainsbury P, Craig J. Consolidated criteria for reporting qualitative research (COREQ): a 32-item checklist for interviews and focus groups. *International Journal for Quality in Health Care*. 2007. Volume 19, Number 6: pp. 349 – 357

No. Item	Guide questions/description	Reported on Page #
**Domain 1: Research team and reflexivity**		
Personal Characteristics		
1. Inter viewer/facilitator	Which author/s conducted the interview or focus group?<br>All	6
2. Credentials	What were the researcher's credentials?<br>ST - PhD in Applied Linguistics<br>JL - PhD in Applied Linguistics<br>AT - PhD in Nursing<br>JT - PhD in Nursing<br>EP - MA in Applied Linguistics	
3. Occupation	What was their occupation at the time of the study?<br>ST - Lecturer in English<br>JL - Professor of Applied Linguistics<br>JT - Professor of Nursing<br>AT - Professor of Nursing<br>EP - Research assistant and postgrad student	
4. Gender	Was the researcher male or female?<br>All female	
5. Experience and training	What experience or training did the researcher have?<br>*All researchers trained in sensitive interviewing techniques*<br>*JL - experience of interviewing women who had experienced pregnancy loss*	
Relationship with participants		
6. Relationship established	Was a relationship established prior to study commencement?	5

For peer review only

	No	
7. Participant knowledge of the interviewer	What did the participants know about the researcher?<br>Place of work<br>Profession<br>Broad aims of the study	5
8. Interviewer characteristics	What characteristics were reported about the interviewer/facilitator?	Name, gender, place of work

**Domain 2: study design**		
Theoretical framework		
9. Methodological orientation and Theory	What methodological orientation was stated to underpin the study?<br>*Thematic analysis involving metaphor*	6
Participant selection		
10. Sampling	How were participants selected? e.g. purposive, convenience, consecutive, snowball.<br>*Purposive; advertisements were shared by relevant charities. Participants self-selected.*	5
11. Method of approach	How were participants approached? e.g. face-to-face, telephone, mail, email-<br>*Recruitment through charities*	5
12. Sample size	How many participants were in the study?<br>*24 across 21 interviews*	7
13. Non-participation	How many people refused to participate or dropped out? Reasons?<br>*0 - participants self-selected*	7
Setting		
14. Setting of data collection	Where was the data collected?<br>*Face to face: In participants' homes or at the TCT HQ in London.*<br>*Online: Skype or Zoom*	6
15. Presence of non-participants	Was anyone else present besides the participants and researchers?<br>*No*	6
16. Description of sample	What are the important characteristics of the sample? e.g. demographic data, date<br>*All participants over 18 and having experienced child bereavement. Further*	

For peer review only

	demographic data not collected as not deemed necessary for study.	
Data collection		
17. Interview guide	Were questions, prompts, guides provided by the authors? Was it pilot tested? *Suggested open-ended interview prompts were provided; not piloted.*	6
18. Repeat interviews	Were repeat interviews carried out? If yes, how many? *No*	6
19. Audio/visual recording	Did the research use audio or visual recording to collect the data? *Audio recorded. Recordings on Skype/Zoom included video data which was destroyed after collection.*	6
20. Field notes	Were field notes made during and/or after the interview or focus group? *Yes*	6
21. Duration	What was the duration of the interviews or focus group? *Dependent on participant - range of 34 to 129 mins*	6
22. Data saturation	Was data saturation discussed? No	n/a
23. Transcripts returned	Were transcripts returned to participants for comment and/or correction? Yes	6
**Domain 3: analysis and findings**		
Data analysis		
24. Number of data coders	How many data coders coded the data? *3*	
25. Description of the coding tree	Did authors provide a description of the coding tree? yes	Online supplementary
26. Derivation of themes	Were themes identified in advance or derived from the data? *Derived from the data*	7
27. Software	What software, if applicable, was used to manage the data? *NVivo*	6
28. Participant checking	Did participants provide feedback on the findings? *No*	

For peer review only

Reporting		
29. Quotations presented	Were participant quotations presented to illustrate the themes/findings? Was each quotation identified? e.g. participant number<br><br>*Quotes identified by participant code*	7-10
30. Data and findings consistent	Was there consistency between the data presented and the findings?<br>*Yes*	
31. Clarity of major themes	Were major themes clearly presented in the findings?<br>*Yes*	
32. Clarity of minor themes	Is there a description of diverse cases or discussion of minor themes?<br>*No*	n/a

For peer review only

BMJ Open

Metaphors that shape parents' perceptions of effective communication with healthcare practitioners following child death: a qualitative UK study

Journal:	*BMJ Open*
Manuscript ID	bmjopen-2021-054991.R1
Article Type:	Original research
Date Submitted by the Author:	22-Nov-2021
Complete List of Authors:	Turner, Sarah; Coventry University, Faculty of Arts and Humanities<br>Littlemore, Jeannette; University of Birmingham, English Language and Linguistics<br>Taylor, Julie; University of Birmingham, School of Nursing, College of Medical and Dental Sciences, ; Birmingham Women's and Children's NHS Foundation Trust,<br>Parr, Eloise; University of Birmingham Edgbaston Campus<br>Topping, AE; University of Birmingham College of Medical and Dental Sciences, School of Nursing; University Hospitals Birmingham NHS Foundation Trust,
**Primary Subject Heading**:	Qualitative research
Secondary Subject Heading:	Emergency medicine, Oncology, Palliative care, Qualitative research
Keywords:	Paediatric oncology < PAEDIATRICS, Paediatric A&E and ambulatory care < PAEDIATRICS, Paediatric palliative care < PAEDIATRICS

SCHOLARONE™
Manuscripts

I, the Submitting Author has the right to grant and does grant on behalf of all authors of the Work (as defined in the below author licence), an exclusive licence and/or a non-exclusive licence for contributions from authors who are: i) UK Crown employees; ii) where BMJ has agreed a CC-BY licence shall apply, and/or iii) in accordance with the terms applicable for US Federal Government officers or employees acting as part of their official duties; on a worldwide, perpetual, irrevocable, royalty-free basis to BMJ Publishing Group Ltd ("BMJ") its licensees and where the relevant Journal is co-owned by BMJ to the co-owners of the Journal, to publish the Work in this journal and any other BMJ products and to exploit all rights, as set out in our licence.

The Submitting Author accepts and understands that any supply made under these terms is made by BMJ to the Submitting Author unless you are acting as an employee on behalf of your employer or a postgraduate student of an affiliated institution which is paying any applicable article publishing charge ("APC") for Open Access articles. Where the Submitting Author wishes to make the Work available on an Open Access basis (and intends to pay the relevant APC), the terms of reuse of such Open Access shall be governed by a Creative Commons licence – details of these licences and which Creative Commons licence will apply to this Work are set out in our licence referred to above.

Other than as permitted in any relevant BMJ Author's Self Archiving Policies, I confirm this Work has not been accepted for publication elsewhere, is not being considered for publication elsewhere and does not duplicate material already published. I confirm all authors consent to publication of this Work and authorise the granting of this licence.

For peer review only

Metaphors that shape parents’ perceptions of effective communication with healthcare practitioners following child death: a qualitative UK study

Title Page

1 Dr Sarah Turner[1,*] sarah.turner@coventry.ac.uk Lecturer in English (Stylistics) https://orcid.org/0000-0003-3414-6828

2 Professor Jeannette Littlemore[2] j.m.littlemore@bham.ac.uk Professor of Applied Linguistics https://orcid.org/0000-0003-4670-0275

3 Professor Julie Taylor[3,4,§] j.taylor.1@bham.ac.uk Professor of Child Protection https://orcid.org/0000-0002-7259-0906

4 Eloise Parr[2] ebp969@student.bham.ac.uk Research Assistant https://orcid.org/0000-0002-7415-5991

5 Professor Annie Topping[3,5] a.e.topping@bham.ac.uk Professor of Nursing https://orcid.org0000-0002-0111-2341

[1]School of Humanities, Coventry University, Coventry, UK

[2]Department of English Language and Linguistics, University of Birmingham, Birmingham, UK

[3] School of Nursing, University of Birmingham, Birmingham, UK

[4] Birmingham Women’s and Children’s Hospitals NHS Trust, Birmingham, UK

[5] University Hospitals Birmingham NHS Foundation Trust, Birmingham, UK

*Lead author address: School of Humanities, Coventry University, Coventry, UK

[§] Corresponding author address: College of Medical and Dental Science, University of Birmingham, Edgbaston, B15 2TT, UK

For peer review only - http://bmjopen.bmj.com/site/about/guidelines.xhtml

Metaphors that shape parents' perceptions of effective communication with healthcare practitioners following child death: a qualitative UK study

Abstract

Objectives To offer an interpretation of bereaved parents' evaluations of communication with healthcare practitioners surrounding the death of a child.

Design Interpretative qualitative study employing thematic and linguistic analyses of metaphor embedded in interview data.

Setting England and Scotland.

Participants 24 bereaved parents (21 women, 3 men)

Methods Participants were recruited through the True Colours Trust website and mailing list, similar UK charities, and word of mouth. Following interviews in person or via video-conferencing platforms (Skype/Zoom), transcripts first underwent thematic and subsequently linguistic analyses supported by Nvivo. A focused analysis of metaphors used by the parents was undertaken allow in-depth interpretation of how they conceptualised their experiences.

Results The findings illuminate the ways parents experienced communication with healthcare practitioners surrounding the death of a child. Key findings from this study suggest that good communication with HCPs following the death of a child should acknowledge parental identity (and that of their child as an individual) and offer opportunities for them to enact this; taking account their emotional and physical experiences; and accommodate their altered experiences of time.

Conclusions This study suggests that healthcare practitioners when communicating with bereaved parents need to recognise, and seek to comprehend, the ways in which the loss impacts upon an individual's identity as a parent, the 'physical' nature of the emotions that can be unleashed, and the ways in which the death of a child can alter their metaphorical conceptions of time.

Keywords bereavement, communication, metaphor, child death, healthcare professionals, parents

Strengths and limitations of the study

- This interpretative qualitative study contributes to the growing literature on the experience of parental bereavement through its focus on the use of metaphor in parents' accounts of child loss.

For peer review only - http://bmjopen.bmj.com/site/about/guidelines.xhtml

- The metaphor analysis afforded a focus not only on the content of the interviews, but also on the language that was used to express this content, providing more depth and nuance to the interpretation of the findings.
- Participants were all UK-based and self-selecting which could limit transferability.

For peer review only

For peer review only - http://bmjopen.bmj.com/site/about/guidelines.xhtml

Metaphors that shape parents' perceptions of effective communication with healthcare practitioners following child death: a qualitative UK study.

INTRODUCTION

When parents experience the death of a child, no matter the age, they experience a range of complex, often intolerably painful emotions[1]. The experience and effects of the death of a child on parents has received considerable attention, including the psychosocial impacts experienced as a consequence of devastating and enduring grief[2]; the experience of parents around end of life of a child[3–5]; what they want and expect from healthcare practitioners (HCPs)[2,6]; and impacts of bereavement services and interventions[1,7]. Less work has focused on the communication remembered by parents as part of their experiences of care or services received, or how that communication subsequently scaffolds their experience of bereavement. However, negative interactions with HCPs following the death of a child have been shown to exacerbate the trauma experienced by the parents[8], while positive interactions such as empathy and provision of information can mitigate it[9,10]. A better understanding of the impact of bereavement and the ways in which it affects parents' evaluations of the communication they had with HCPs in the period surrounding the death may enable design of structures, processes, interventions and training to improve individualised compassionate bereavement care.

BACKGROUND

The death of a child engenders a range of intense and complex emotions that may be difficult to articulate[11]. The loss of a child has been associated with poorer parental physical health and increased mortality[12]. Staff regularly providing end-of life care also experience high levels of emotional exhaustion[5]. These are further compounded when HCPs feel guilt about inadequate, or missed care, poor communication and/or contain expressions of grief or emotions in order to protect families from further distress[13]. Openness, a preparedness to talk about the bereavement, an ability to listen, and the avoidance of unwarranted advice [14,15] are characteristics that have been identified as components of effective communication to date.

Staff involved in managing deaths occurring in healthcare settings have well practiced roles and responsibilities. Sociocultural values and beliefs about death and dying are implicitly embedded in the organisation of services and inevitably demonstrated in the behaviours, attitudes and discourses used by the staff involved[16]. This is most evident in what could be interpreted as a proceduralisation of dying[17,18] which can exclude the individual who is dying and parents and family members experiencing the loss. The well rehearsed concept of a good death is less well understood in relation to the death of a child or young person[19]. Possibly as greater emphasis is placed on dying and/or bereavement rather than the period surrounding death, and different participants (patients, family/parents, and staff) may have

For peer review only - http://bmjopen.bmj.com/site/about/guidelines.xhtml

different needs and requirements for control and participation/non-participation in the social context where the dying and death are enacted.

Parents provide mixed accounts of the care that they recall having received in the period surrounding the death of their child[2,20] with some reporting highly positive experiences of compassionate care and others less positive accounts. What is clear is parents' value open and honest communication with HCPs, want to be actively involved, and their emotional distress acknowledged. Ideally they want to communicate with HCPs with whom they have already developed established and trusting relationships[4]. Furthermore, they want to say goodbye, to understand why their child died and to receive follow up care from HCPs involved in the care of their child[2]. Ensuring these standards are achieved may be particularly challenging in the case of sudden death or in emergency, trauma and critical care contexts. This means that good guidance for healthcare practitioners on how best to communicate with parents following the death of their child is essential.

In this study, we focused on the experiences of parents in the immediate aftermath of the death of their child, and their recollected communications with HCPs. We included parents of both adult and non-adult children. The term 'healthcare practitioners' (HCPs) is used in a broad sense to include all of those who come into contact with the parents at the hospital, not just the doctors and nurses.

METHODS

Aim

The aim of this research was to investigate how parents' experiences of bereavement and child death shaped their perceptions of the communication they had with HCPs. In this paper, we consider 'communication' to encompass both language and behaviour, and the interactions between actors.

By gaining insights into parents' experiences of child death, we sought to better understand what constitutes effective communication, with a view to improving care after loss.

Design and Setting

We used an interpretative descriptive qualitative design[21]. The study is reported using Consolidated criteria for Reporting Qualitative research guidelines (see online supplementary file).

Participants

Our participants comprised parents aged at least 18 years who had lost a child of any age, who could speak English at conversational level, and who were able to provide informed consent.

Sampling and recruitment

A purposive sampling procedure was employed. Recruitment adverts were posted by the True Colours Trust, Compassionate Friends, and other similar charities (e.g. Acorns Children's

For peer review only - http://bmjopen.bmj.com/site/about/guidelines.xhtml

Hospice, Winston's Wish etc). Potential participants who responded to the posting were contacted by the research team and invited to participate in an interview.

Data collection

In total, 24 participants were interviewed, in 21 interviews. The majority of the interviews were conducted on a 1-1 basis, with all authors undertaking interviews. On three occasions however we undertook joint interviews with male and female participants at their request (interviews 2, 5 and 20). All interviews involved female participants (n=21). Interestingly just under half of our respondents were themselves HCPs. The interviews were conducted face to face (n=8), or via video-conference platforms (n=13), according to participant preference, location, or COVID-19 restrictions. All but two of the face to face interviews were conducted in participants' homes, except two conducted at the Charity headquarters. Interviews lasted an average of 81 minutes (range: 34-129 mins).

Prior to interview, participants were informed how the findings derived from their interview data would be reported and disseminated in training materials. All participants provided informed consent prior to interview. Interviews were undertaken by all five authors, with some interviewers working together. A topic guide was developed to serve as a framework for the semi-structured interviews (see box 1). Open questions were used to encourage extended answers, and to allow the interviewer(s) to elicit further detail on points of interest.

Box 1 Sample of interview prompts
• Can you tell us about [name of child]?<br>• Please could you describe what happened ….?<br>• Please could you tell me about what happened next…?<br>• How did you feel about…?<br>• Can you tell me about the communication you had with the healthcare practitioners?<br>• Can you recall any examples of particularly sensitive and effective communication you received?<br>• Can you recall any examples of particularly insensitive communication you received?<br>• Imagine you are talking to someone who supports people who have had an experience like yours. What advice would you give them?<br>• What would you like to see changed?

Audio recordings were made of the interviews with consent. In the case of video-conference interviews, recordings included video data, but these were destroyed and only audio recordings retained.

Ethical considerations

Given the sensitive nature of the topic, we received full ethical approval from the University of Birmingham [ERN_19-1582] and had a range of distress protocols and appropriate safeguarding measures in place if required. The multidisciplinary research team (both clinical

For peer review only - http://bmjopen.bmj.com/site/about/guidelines.xhtml

and linguistic) is well rehearsed and trained in dealing with sensitive topics and vulnerable populations. We did not encounter any unexpected ethical dilemmas.

Analysis

Audio recordings were transcribed verbatim and all identifying information removed. Transcripts were sent to the participants to allow them to make any corrections or additions they felt necessary. Three participants requested minor changes that would preserve anonymity and we acceded to all requests. Initial peer checking of codes derived from the interview transcripts was undertaken by the entire team, then fully coded by ST, JL and EP using Nvivo. Any discrepancies were resolved through discussion with the whole team. We found that Nvivo allowed us to develop a complex coding scheme on multiple levels and to cross reference coding categories.

We first undertook a thematic analysis [22] to identify parents' experiences of bereavement and communication. A full list of identified themes is provided in the online supplementary file 1]. Subsequently, we combined our thematic analysis with a metaphor analysis which enabled us to explore how parents conceptualised these experiences. The metaphor analysis involved coding the language used by participants relating to each of the categories for metaphor. This focus on metaphors was designed to provide deeper insights into the nature of the experience, and explore the extent to which parents' perceptions of communication could be explained by these experiences.

In order to identify metaphor in the transcripts, we used Cameron and Maslen's Vehicle Identification Procedure[23]. This analytic procedure operates at the level of the phrase and involves recognition of the fact that something is being described in terms of a different entity.

Patient and Public Involvement

The study engaged with bereaved families throughout. It was designed in communication with the True Colours Trust who funded the research, and who work closely with bereaved parents. Study results will be shared with all participants and ultimately integrated in training materials.

FINDINGS

Participants

Twenty-four people participated in 21 interviews. Table 1 gives further information about the circumstances of death in each interview.

Table 1: Circumstances of death

Interview number	Age of child (years)	Cause of death	When death occurred
1	0-5	Road traffic collision	<5yrs
2	0-5	Illness (sudden)	>5yrs

For peer review only - http://bmjopen.bmj.com/site/about/guidelines.xhtml

3	16-20	Illness (sudden)	>5yrs
4	0-5	Illness (sudden)	>5yrs
5	16-20	Drug-related death	<5yrs
6	0-5	Illness (sudden)	<5yrs
7*	0-5	Illness (genetic condition)	>5yrs + <5yrs
8	0-5	Illness (sudden)	>5yrs
9	16-20	Suicide	>5yrs
10	21-30	Suicide	>5yrs
11	16-20	Drug-related death	<5yrs
12	21-30	Unknown (ongoing inquiry)	<5yrs
13	21-30	Suicide	<5yrs
14	21-30	Illness (cancer)	<5yrs
15	16-20	Road traffic collision	>5yrs
16	21-30	Murder	<5yrs
17	0-5	Illness (sudden)	<5yrs
18	0-5	Illness (sudden)	>5yrs
19	0-5	Illness (genetic condition)	>5yrs
20	0-5	Illness (sudden)	<5yrs
21	0-5	Illness (sudden)	>5yrs

* interviewee discusses two child deaths associated with the same inherited condition

In all our interviews, we found that the parents had vivid memories of some parts of the peri-death experience whereas others were more hazy. In all cases, the ways in which HCPs communicated with the parents during this period played a crucial role in shaping the experience. In this example, the parent uses metaphor to illustrate the painful impact of the doctor's words:

> years later, I can remember every word. I can even remember his tone of voice as he said it. Absolutely etched with acid into my brain
> [Interview 3]

For peer review only - http://bmjopen.bmj.com/site/about/guidelines.xhtml

Through our thematic and metaphorical analyses we identified 14 categories, which included different kinds of communication, people involved, and experiences surrounding events associated with the loss. These are shown in Table 1 in the supplementary file.

To answer our research questions we examined the metaphors within these categories in order to identify overarching themes in the data where metaphor was playing an important role. The three themes reported here and shown in Figure 1 are: (1) Identity (2) Emotional and Physical Response and (3) Time.

[Figure 1]

Figure 1: Metaphorical Themes

For peer review only

Identities of parents and children

Parents appeared to struggle with the loss not only as the death of the child but also as a significant threat to their identities as parents. Some parents talked about how leaving their child in the hospital after death was made more difficult, as it constituted 'leaving them behind'. Parents felt that they still needed to look after their child, as one parent explained how she was 'distraught' that her daughter was 'shut up' in the mortuary as she was claustrophobic. These utterances reflect a level of metaphoric thinking in which parents conceptualise their child as being, on some level, still alive. Here a bereaved parent talked about the excellent care received when they went to identify their son's body:

> [a member of staff at the mortuary] crouched down, she took my hand and said 'hello NAME, I've got your little boy and I'm keeping him safe, do you want to come and see him?' [Interview 5]

Many of the parents commented on how they were given the time and space to act in ways that seemed right to them as it respected their child's identity and theirs as parents. One couple (Interview 2) appreciated how the consultant arranged for their child's body to be taken to a children's hospice, where it could be kept in a refrigerated room that had been decorated like a child's bedroom.

Conversely, some parents reported examples of care and communication that failed to recognise their child as an individual. This parent describes the distress she felt when hospital staff attempted to fit her infant into a Moses basket:

> They tried to put him in a Moses basket cos they thought it was a cot death, they obviously didn't say the age on the phone call across [...] They probably should know about the age of the child, that was really – cos they did try and like squeeze him in in a way as well. So even when – you could clearly see he wasn't gonna fit in that. I just thought that was quite upsetting. Like he wouldn't have been in a Moses basket at that age anyway [Interview 2].

This was particularly distressing to the parents because it seemed that the HCPs were more concerned with following the procedure rather than with responding flexibly to the reality of

For peer review only - http://bmjopen.bmj.com/site/about/guidelines.xhtml

the situation. This contributed to the parents' impression that the care their child received was a "one size fits all" rather than tailored to the individual needs of their child.

In some cases, parents were told that they were not allowed to interact with their child following the death because the circumstances of the death were unknown. Although necessary, it caused extreme distress, exacerbated by insufficient explanations and/or management of parental expectations. Another couple (Interview 6) spoke of the 'horrific' experience of having to take a service lift down to the 'old-fashioned' and 'cold' mortuary. The parents felt that they were insufficiently prepared for what they would encounter, and their role as parents was discounted to that of observers of a body.

Emotional and Physical Responses

A second theme related to the 'physical' nature of emotions unleashed by the death of a child. Parents often referred to these experiences in metaphorical ways conveying the strength of their emotional response to the death.

> You're frozen, you're like a zombie, you just can't, everything's frozen up inside you [Interview 3]

> It's like you're full of cotton wool in your head and people do have to keep telling you things over and over and over again … so just kind of time, don't be surprised you've got a woolen brain sitting in front of you
>
> [Interview 3]

These quotes emphasise the effect death has and how HCPs may need to recognise this and in terms of ability of parents to receive information. In the following example, a parent talks about how she had kept a bereavement support leaflet from the hospital for some months following her daughter's death.

> It was there in the booklet that HOSPITAL had given me [...] my attention was directed to it and I hadn't noticed it. I'd been to my doctor and he'd given me sleeping tablets and I wasn't taking them, I was saving them up. It was stupid, I know it was stupid. [I was] sitting with all these tablets and a bottle of wine and just thinking, 'I can't do this'. … And I thought I'm gonna have to get help and I knew the Samaritans number was in that booklet they'd given me. So I'd found the booklet, found the Samaritans and then just underneath I saw Compassionate Friends. And so I phoned the helpline … and got an answerphone, 'We'll get back to you' and I thought, 'I bet they don't', but they did. And I spoke to a lady called NAME ... and she was great. She'd lost her daughter about 14 or 15 years before and just hearing her say that she'd survived more than anything else helped me.
>
> [Interview 3]

At times parents employed metaphor to express the fact that they no longer wanted to engage with the world:

For peer review only - http://bmjopen.bmj.com/site/about/guidelines.xhtml

> We just wanted to hide. Literally had this sense of 'I wanna get a cave, I don't wanna see the world, I don't wanna engage with anybody or anything'. I can't function with the world being normal. I nearly always wore contact lenses, I rarely wore glasses but [then] I wore glasses on purpose so that I could just take them off half the time, cos I just wanted everything to go away [Interview 4].

She draws a metaphorical comparison between the act of seeing and the act of engaging with the world around her, which allows her to take control of her situation by literally removing her glasses, thus enabling her to metaphorically withdraw from the world.

At times, parents commented that HCPs appeared not to take sufficient account of the fact that appearing calm concealed the depth of their distress, how staff mistook calmness for strength and indeed congratulated parents for coping. Whereas the bereaved felt under pressure to present a 'brave face':

> Throughout the early days people (hospital staff etc.,) would constantly say how strong and brave I was. I kept saying " no I'm not, I'm just pretending", but eventually I felt that I had to be this "brave, strong" woman or I would be letting everyone down. If I could say one thing to them [hospital staff] it would be to ask them not to say that. We are not brave and strong, just in a state of severe shock and watching ourselves go through the motions and it's really unhelpful to make us feel we must behave in a certain way [Interview 11].

By praising parents HCPs were inadvertently reinforcing the idea that following bereavement, some kinds of behaviour tend to be more acceptable than others and that overt emotional displays are to be discouraged[4].

Disordered Perceptions of Time

Some of the parents in our study reported having experienced disordered perceptions of time, and the passing of time was often foregrounded. This is demonstrated in the following examples:

> So suddenly … I remember thinking, 'Slow down! It's like I still don't know what's happened,' and then suddenly, we were like, 'Whoo!' and we were heading towards an inquest but having said that, it was a year, this whole process ... so it wasn't slow down time. It was just - so we had no control [Interview 1].

> when you've had such a massive shock like that, it just takes so long for your brain to catch up [Interview 1].

Disordered time appeared to influence how interactions with HCPs were perceived. For example what could be viewed as acts of caring for were interpreted as simply procedural and are therefore less effective. In some cases, this may prevent parents from reaping any benefit from the care provided. For example, one participant described receiving a memory box without any (remembered) explanation of what it was, or what was in it, and did not dare to open it:

> At that point, a nurse put something in my mum's hands and it was inside a cardboard box and just said, 'Here's his memory box'. So my mum was a bit like, 'Thanks'. It was horrific for her. So she walked out, there was no other explanation of it and we

For peer review only - http://bmjopen.bmj.com/site/about/guidelines.xhtml

> walked out and she just put it on the dining table when we got home. And like for days after everyone was like, 'What's in that box?', but no one dared open it because - my mum was saying, 'They said oh it's his memory box, I didn't want to open it in case it's his things in there' [Interview 6].

We see from this example that what may have started out as an enactment of good practice in bereavement support - the giving of a memory box - had become for this parent, something to be feared due to the way in which it was presented.

DISCUSSION

The findings from this study suggest that good communication with HCPs following the death of a child should acknowledge parental identity (and that of their child as an individual) and offer opportunities for them to enact this; taking account their emotional and physical experiences; and accommodate their altered experiences of time. Practitioners need to acknowledge the individual needs of the parents while working within the parameters of best practice and legal considerations[18,24].

Identity

In keeping with previous research[25,26], we found that many of the parents reported a strong and sustained bond with their child, which led to a continuing need to 'parent' their child. The importance of these bonds may be understood as a response to the threat to parental identity that the loss of a child represents. Thus parents experiencing their child's death are in part losing their role as a parent [27]. Parents in our study highlighted the importance of being able to spend time with their child following death and to continue enacting their roles as parents[28–30]. The opportunity to do this allows for a 'slow and gentle separation' at a pace set by the parents, reduced feelings of helplessness, and the potential to form 'bittersweet memories' which may later represent a source of comfort[28,29]. The importance of protected time within the grief process particularly spending time with the child's body represents symbolically privileged status of parent and other family members in death[31]. This also chimes with broader conceptualisations of effective communication that emphasise openness and the need not to withdraw from the bereaved[14,15].

The behaviour of parents towards their child's body can be understood as an example of metaphorical thinking, where the child is perceived as needing the same care as in life. This involves a blurring of the boundaries between life and death where parents enact behaviours they would have engaged in if the child were alive. Our study provides further evidence of the importance of opportunities to engage in these behaviours, and their facilitative role in the grieving process.

HCPs have an important role to play in acknowledging bereaved parents' continuing bonds with their children, and facilitating opportunities for parents to perform these behaviours. The parents in our study made frequent references to cases where HCPs were either successful in doing so, or where they felt the care they received was lacking in this regard. This was often a determining factor in their evaluation of the effectiveness of the overall care that they had received. For example, parents in our study appreciated instances of HCPs treating their child's body with respect and dignity. This was represented in our findings describing HCPs speaking to children after death as a means of respecting the child's presence and personhood[32].

For peer review only - http://bmjopen.bmj.com/site/about/guidelines.xhtml

Effective linguistic and behavioural communication therefore involves recognising that the loss of a child represents a significant threat to parental identity, and that parents should be given the time, space and opportunity to engage in activities that enable them to enact that identity. Similarly, there is a need to understand the continuing bonds that the parents still have with their child, even when these may be appear in ways that seem unusual.

Emotional and physical responses

The parents in our study drew heavily on embodied metaphors to describe the strength and impact of the bereavement and ensuing emotions. The metaphors used had such a strong physical basis is in line with other studies that propose powerful, embodied metaphors can be the only device strong enough to express emotions following traumatic experiences[33,34,34–37]. Although metaphor is sometimes seen as a poetic device, operating solely at the level of language, we have demonstrated that it is inappropriate to see it as a surface-level linguistic phenomenon, particularly in the context of traumatic experiences. The physical nature of the metaphors that bereaved parents employ when describing the intensity of their experiences and the fact that these metaphors sometimes spill over into their behaviour (e.g. the parent who literally removed her glasses) provide powerful insights into their physical and emotional state and as such are not simply 'figures of speech'. These observations are also in line with previous research that has demonstrated that grief and emotional trauma may manifest as physical sensations[38].

Effective care and communication following child death takes account of the all-encompassing, frequently embodied nature of grief, and recognises that this may have an effect on parents' abilities to make decisions and understand the meaning communicated in the care provided. This is in line with previous research showing that effective communication following bereavement needs to take account of the bereaved state of mind [39]. Grief may manifest in behaviours that appear unusual or may go unnoticed (removing glasses), but are motivated by highly individualised metaphorical thinking patterns that represent the parents' attempts to manage and come to terms with their experiences.

Disordered Perceptions of Time

Following the death of a child, we found that parents sometimes experienced disordered perceptions of time. Trauma can exert a strong effect on perceptions of time, reporting the phenomena of time stopping, slowing down, speeding up or becoming irrelevant[40]. All of these descriptions draw on the metaphorical relationship between time and movement through space[41]. Time might metaphorically expand or contract, or do both at the same time, and parents may feel that they are no longer part of the same 'reality' as those around them. While altered perceptions of time over the course of the grieving process have been attested to in narratives following child loss[42], our findings indicate that changed experiences of time in the immediate aftermath of a loss may also have important implications for care. It is therefore important for HCPs to recognise that parents' emotional state may not be conducive to receiving and processing complex information, and they may need to be given the information more than once and/or in more than one format[33]. HCPs should demonstrate patience and sensitivity to parents' difficulties in processing information.

For peer review only - http://bmjopen.bmj.com/site/about/guidelines.xhtml

There is a danger that care following the death of a child can become procedural, and as a result may not be empathetic to the needs of parents. For example, providing a memory box may constitute excellent bereavement care, but if this is given without clear explanation, or choice, may become a source of anxiety. Following formal procedures without sensitivity to individual needs may result in greater disquiet.

This study has contributed to the growing literature on the experience of parental bereavement but its focus on the use of metaphor in parents' accounts of child loss provides valuable insights. Key aspects of the experience have been identified that need to be considered when providing care for the bereaved. Our metaphorical analysis has afforded a focus on the on the language that was used to express content in qualitative interviews, providing more depth and nuance to interpretation. The number of participants was relatively small UK-based and self-selecting which could limit the transferability of our findings. Future studies could usefully explore metaphor with more participants with different demographic characteristics and in different settings.

CONCLUSION

This study has shown how the analysis of metaphors used by parents when describing their experiences of bereavement can provide a deep and nuanced understanding of their experiences and how these experiences affect their evaluations of the communication they receive from HCPs. We have demonstrated that effective communication and strategies to ameliorate distress entails an understanding of the feelings and experiences of the bereaved and takes these into account. Conversely, ineffective communication is not grounded in this awareness.

Effective communication that takes account of parents' experiences of bereavement may involve:

Identity:

- Demonstrating an awareness and acknowledgement of the parents' identities as parents, and the child's personhood
- Demonstrating respect for the child's body and reassure parents that the child is being cared for even after death
- Facilitating opportunities for the parents to enact their roles as parents

Emotional and Physical Responses

- Being aware that the all-encompassing nature of the grief may render parents unable to process and act upon information
- Recognising that parents may engage in individualised behaviours that may seem unusual or go unrecognised as enactments of grief.

Time

- Recognising that parents may be experiencing time in altered ways, and that this may have an impact on their ability to process information.
- Explaining information in a clear but sensitive way, recognising that information may need to be given more than once or in different formats.

For peer review only - http://bmjopen.bmj.com/site/about/guidelines.xhtml

- Demonstrating patience, giving parents time and not rushing them through the various procedures or difficult decisions.

References

1 Donovan LA, Wakefield CE, Russell V, *et al.* Hospital-based bereavement services following the death of a child: a mixed study review. *Palliative medicine* 2015;**29**:193–210.

2 Garstang J, Griffiths F, Sidebotham P. What do bereaved parents want from professionals after the sudden death of their child: a systematic review of the literature. *BMC pediatrics* 2014;**14**:1–17.

3 Ekberg S, Bradford N, Herbert A, *et al.* Healthcare users' experiences of communicating with healthcare professionals about children who have life-limiting conditions: a qualitative systematic review protocol. *JBI Evidence Synthesis* 2015;**13**:33–42.

4 Ekberg S, Bradford NK, Herbert A, *et al.* Healthcare users' experiences of communicating with healthcare professionals about children who have life-limiting conditions: a qualitative systematic review. *Journal of palliative medicine* 2018;**21**:1518–28.

5 McConnell T, Porter S. The experience of providing end of life care at a children's hospice: a qualitative study. *BMC palliative care* 2017;**16**:1–6.

6 Tenzek KE, Depner R. Still searching: a meta-synthesis of a good death from the bereaved family member perspective. *Behavioral Sciences* 2017;**7**:25.

7 Kochen EM, Jenken F, Boelen PA, *et al.* When a child dies: a systematic review of well-defined parent-focused bereavement interventions and their alignment with grief-and loss theories. *BMC palliative care* 2020;**19**:1–22.

8 Janzen L, Cadell S, Westhues A. From death notification through the funeral: Bereaved parents' experiences and their advice to professionals. *Omega-Journal of Death and Dying* 2004;**48**:149–64.

9 Meert KL, Briller SH, Myers Schim S, *et al.* Examining the needs of bereaved parents in the pediatric intensive care unit: a qualitative study. *Death studies* 2009;**33**:712–40.

10 Price J, Jordan J, Prior L, *et al.* Living through the death of a child: A qualitative study of bereaved parents' experiences. *International journal of nursing studies* 2011;**48**:1384–92.

11 Arnold J, Gemma PB. The continuing process of parental grief. *Death studies* 2008;**32**:658–73.

12 Endo K, Yonemoto N, Yamada M. Interventions for bereaved parents following a child's death: A systematic review. *Palliative Medicine* 2015;**29**:590–604.

13 Papadatou D, Bellali T, Papazoglou I, *et al.* Greek nurse and physician grief as a result of caring for children dying of cancer. *Pediatric nursing* 2002;**28**:345–54.

For peer review only - http://bmjopen.bmj.com/site/about/guidelines.xhtml

14 Dyregrov K. Micro-sociological analysis of social support following traumatic bereavement: Unhelpful and avoidant responses from the community. *Omega-Journal of Death and Dying* 2004;**48**:23–44.

15 Holmberg L. "Words that made a difference": Communication in bereavement. *Journal of loss and trauma* 2007;**12**:11–31.

16 Cheek J. At the margins? Discourse analysis and qualitative research. *Qualitative health research* 2004;**14**:1140–50.

17 Cottrell L, Duggleby W. The" good death": An integrative literature review. *Palliative & supportive care* 2016;**14**:686.

18 National Institute of Clinical Excellence. End of life care for infants, children and young people with life-limiting conditions: planning and management NICE guideline. 2016.www.nice.org.uk/guidance/ng61

19 Chong PH, Walshe C, Hughes S. Perceptions of a good death in children with life-shortening conditions: an integrative review. *Journal of palliative medicine* 2019;**22**:714–23.

20 Butler A, Hall H, Willetts G, *et al.* Parents' experiences of healthcare provider actions when their child dies: an integrative review of the literature. *Journal for specialists in pediatric nursing: JSPN* 2014;**20**:5–20.

21 Thorne S. Interpretative Description. In: Beck CT, ed. *Routledge International Handbook of Qualitative Nursing Research*. Routledge 2013. 295–306.

22 Braun, V., Clarke, V. Thematic Analysis. In: *APA Handbook of Research Methods in Psychology: Vol. 2. Research Designs.* New York: : American Psychological Association 2012. 57–71.

23 Cameron L, Maslen R. Metaphor analysis. *London: Equinox* 2010.

24 National Quality Board. Learning from Deaths Guidance for NHS trusts on working with bereaved families and carer. https://www.england.nhs.uk/wp-content/uploads/2018/08/learning-from-deaths-working-with-families-v2.pdf

25 Harper M, O'Connor R, Dickson A, *et al.* Mothers continuing bonds and ambivalence to personal mortality after the death of their child–an interpretative phenomenological analysis. *Psychology, Health & Medicine* 2011;**16**:203–14.

26 Toller PW. Bereaved parents' negotiation of identity following the death of a child. *Communication Studies* 2008;**59**:306–21.

27 Klass D. *Parental grief: Solace and resolution*. Springer Publishing Co 1988.

28 Davies R. Mothers' stories of loss: their need to be with their dying child and their child's body after death. *Journal of Child Health Care* 2005;**9**:288–300.

29 Forrester L. Bereaved parents' experiences of the use of 'cold bedrooms' following the death of their child. *International journal of palliative nursing* 2008;**14**:578–85.

For peer review only - http://bmjopen.bmj.com/site/about/guidelines.xhtml

30 McGraw SA, Truog RD, Solomon MZ, *et al.* "I was able to still be her mom" Parenting at end of life in the PICU. *Pediatric critical care medicine: a journal of the Society of Critical Care Medicine and the World Federation of Pediatric Intensive and Critical Care Societies* 2012;**13**:e350.

31 Norton EA, Mastroyannopoulou K, Rushworth I. Parents experience of using "cold" facilities at a children's hospice after the death of their baby: A qualitative study. *Death Studies* 2020;:1–7.

32 Forster EM, Windsor C. Speaking to the deceased child: Australian health professional perspectives in paediatric end-of-life care. *International journal of palliative nursing* 2014;**20**:502–8.

33 Littlemore J, Turner S. What Can Metaphor Tell Us About Experiences of Pregnancy Loss and How Are These Experiences Reflected in Midwife Practice? *Frontiers in Communication* 2019;**4**:42.

34 Turner S, Littlemore J, Fuller D, *et al.* The production of time-related metaphors by people who have experienced pregnancy loss. *Producing Figurative Expression: Theoretical, experimental and practical perspectives* 2020;**10**:389.

35 Gibbs RW, Franks H. Embodied metaphor in women's narratives about their experiences with cancer. *Health communication* 2002;**14**:139–65.

36 Semino E. Descriptions of pain, metaphor, and embodied simulation. *Metaphor and Symbol* 2010;**25**:205–26.

37 Semino E. Metaphor, creativity, and the experience of pain across genres. *Creativity in language and literature: The state of the art* 2011;:83–102.

38 Gilmer MJ, Foster TL, Vannatta K, *et al.* Changes in parents after the death of a child from cancer. *Journal of pain and symptom management* 2012;**44**:572–82.

39 Breen LJ, O'Connor M. Family and social networks after bereavement: Experiences of support, change and isolation. *Journal of Family Therapy* 2011;**33**:98–120.

40 Holman EA, Grisham EL. When time falls apart: The public health implications of distorted time perception in the age of COVID-19. *Psychological trauma: theory, research, practice, and policy* 2020.

41 Moore KE. The spatial language of time. *Amsterdam & Philadelphia: John Benjamins* 2014.

42 Barak A, Leichtentritt RD. Configurations of time in bereaved parents' narratives. *Qualitative Health Research* 2014;**24**:1090–101.

ACKNOWLEDGEMENTS

The authors thank the True Colours Trust for supporting this project.

For peer review only - http://bmjopen.bmj.com/site/about/guidelines.xhtml

CONTRIBUTORS

All authors planned the study, applied for funding and applied for ethical approval. All authors undertook interviews with participants and undertook initial analysis. JL, ST and EP undertook the main thematic analysis, checking any discrepancies with JT and AT. All authors collaborated on the interpretation and implications of the findings. All authors contributed to drafting of the manuscript, which was circulated iteratively for critical revision. All authors approved the final version of the manuscript.

COMPETING INTERESTS

None declared

FUNDING

This project was funded by the True Colours Trust

PATIENT CONSENT FOR PUBLICATION

Obtained

ETHICAL APPROVAL

The study had full ethical approval from University of Birmingham's Ethical Review Committee (ERN_19-1582)

PROVENANCE AND PEER REVIEW

Commissioned and funded by the True Colours Trust; grant number TCT1609; externally peer reviewed

DATA AVAILABILITY STATEMENT

No data are available. The research involved interviews in which bereaved parents gave personal accounts of their experiences. Participants consented to their data being used for this project only. Therefore, we will not share the data for any other purpose.

For peer review only

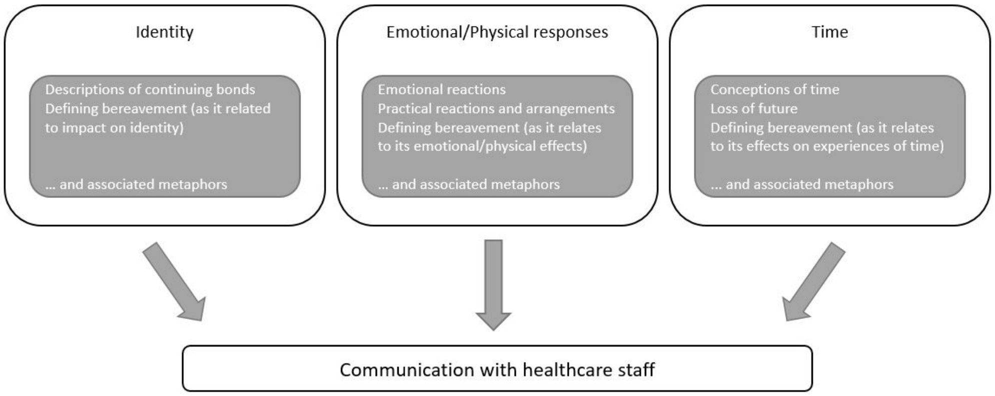

Figure 1: Metaphorical Themes

97x38mm (300 x 300 DPI)

Supplementary material Table 1

Coding categories used in our analysis

01 Bad Communication
- a Dehumanisation of child
- b Lack of empathy
- c Use of jargon
- d Ineffective use of time
- e Wrong information provided
- f Bad advice
- g Failure to communicate key information

02 Good Communication
- a Recognition of child
- b Empathy
- c Clear language
- d Effective use of time
- e Correct information provided
- f Good advice
- g Successful communication of key information

03 Emotional Reactions
- Emotional reactions - negative
- Emotional reactions - neither positive or negative
- Emotional reactions - positive
- Unexpectedly powerful reactions - negative
- Unexpectedly powerful reactions - positive
- Wished for emotional reaction

04 Practical Reactions and Arrangements
- Practical reactions and arrangements - neither positive or negative
- Practical reactions and arrangements - positive
- Practical reactions and arrangements - negative
- Symbolic Behaviours
- Unexpectedly powerful reactions - negative
- Unexpectedly powerful reactions - positive
- Wished for practical reaction

05 People Involved
- GPs
- Hospital staff
- Doctors
- Nurses
- Others - hospital
- Receptionists
- Mortuary staff
- Paramedics

06 Continuing Bonds
07 Loss of Future
08 Defining Bereavement
09 Conceptions of time

For peer review only

10 Metaphors<br>11 Advice that parents would give

For peer review only

Parents' experiences of healthcare professional communication following the death of a child: A qualitative study in the UK

Consolidated criteria for reporting qualitative studies (COREQ): 32-item checklist

Developed from:
Tong A, Sainsbury P, Craig J. Consolidated criteria for reporting qualitative research (COREQ): a 32-item checklist for interviews and focus groups. *International Journal for Quality in Health Care*. 2007. Volume 19, Number 6: pp. 349 – 357

No. Item	Guide questions/description	Reported on Page #
**Domain 1: Research team and reflexivity**		
Personal Characteristics		
1. Inter viewer/facilitator	Which author/s conducted the interview or focus group?<br>All	6
2. Credentials	What were the researcher's credentials?<br>ST - PhD in Applied Linguistics<br>JL - PhD in Applied Linguistics<br>AT - PhD in Nursing<br>JT - PhD in Nursing<br>EP - MA in Applied Linguistics	
3. Occupation	What was their occupation at the time of the study?<br>ST - Lecturer in English<br>JL - Professor of Applied Linguistics<br>JT - Professor of Nursing<br>AT - Professor of Nursing<br>EP - Research assistant and postgrad student	
4. Gender	Was the researcher male or female?<br>All female	
5. Experience and training	What experience or training did the researcher have?<br>*All researchers trained in sensitive interviewing techniques*<br>*JL - experience of interviewing women who had experienced pregnancy loss*	
Relationship with participants		
6. Relationship established	Was a relationship established prior to study commencement?	5

For peer review only

	No	
7. Participant knowledge of the interviewer	What did the participants know about the researcher?<br>Place of work<br>Profession<br>Broad aims of the study	5
8. Interviewer characteristics	What characteristics were reported about the interviewer/facilitator?	Name, gender, place of work

**Domain 2: study design**		
Theoretical framework		
9. Methodological orientation and Theory	What methodological orientation was stated to underpin the study?<br>*Thematic analysis involving metaphor*	6
Participant selection		
10. Sampling	How were participants selected? e.g. purposive, convenience, consecutive, snowball.<br>*Purposive; advertisements were shared by relevant charities. Participants self-selected.*	5
11. Method of approach	How were participants approached? e.g. face-to-face, telephone, mail, email-<br>*Recruitment through charities*	5
12. Sample size	How many participants were in the study?<br>*24 across 21 interviews*	7
13. Non-participation	How many people refused to participate or dropped out? Reasons?<br>*0 - participants self-selected*	7
Setting		
14. Setting of data collection	Where was the data collected?<br>*Face to face: In participants' homes or at the TCT HQ in London.*<br>*Online: Skype or Zoom*	6
15. Presence of non-participants	Was anyone else present besides the participants and researchers?<br>*No*	6
16. Description of sample	What are the important characteristics of the sample? e.g. demographic data, date<br>*All participants over 18 and having experienced child bereavement. Further*	

For peer review only

	demographic data not collected as not deemed necessary for study.	
Data collection		
17. Interview guide	Were questions, prompts, guides provided by the authors? Was it pilot tested? *Suggested open-ended interview prompts were provided; not piloted.*	6
18. Repeat interviews	Were repeat interviews carried out? If yes, how many? *No*	6
19. Audio/visual recording	Did the research use audio or visual recording to collect the data? *Audio recorded. Recordings on Skype/Zoom included video data which was destroyed after collection.*	6
20. Field notes	Were field notes made during and/or after the interview or focus group? *Yes*	6
21. Duration	What was the duration of the interviews or focus group? *Dependent on participant - range of 34 to 129 mins*	6
22. Data saturation	Was data saturation discussed? No	n/a
23. Transcripts returned	Were transcripts returned to participants for comment and/or correction? Yes	6
**Domain 3: analysis and findings**		
Data analysis		
24. Number of data coders	How many data coders coded the data? *3*	
25. Description of the coding tree	Did authors provide a description of the coding tree? yes	Online supplementary
26. Derivation of themes	Were themes identified in advance or derived from the data? *Derived from the data*	7
27. Software	What software, if applicable, was used to manage the data? *NVivo*	6
28. Participant checking	Did participants provide feedback on the findings? *No*	

For peer review only

Reporting		
29. Quotations presented	Were participant quotations presented to illustrate the themes/findings? Was each quotation identified? e.g. participant number<br><br>*Quotes identified by participant code*	7-10
30. Data and findings consistent	Was there consistency between the data presented and the findings?<br>*Yes*	
31. Clarity of major themes	Were major themes clearly presented in the findings?<br>*Yes*	
32. Clarity of minor themes	Is there a description of diverse cases or discussion of minor themes?<br>*No*	n/a

For peer review only
